# Safety and efficacy of pharmacological inhibition of ketohexokinase in hereditary fructose intolerance

**DOI:** 10.1172/JCI187376

**Published:** 2025-02-11

**Authors:** Evi J.C. Koene, Amée M. Buziau, David Cassiman, Timothy M. Cox, Judith Bons, Jean L.J.M. Scheijen, Casper G. Schalkwijk, Steven J.R. Meex, Aditi R. Saxena, William P. Esler, Vera B. Schrauwen-Hinderling, Patrick Schrauwen, Martijn C.G.J. Brouwers

**Affiliations:** 1 Department of Nutrition and Movement Sciences and; 2 Institute of Nutrition and Translational Research in Metabolism (NUTRIM), Maastricht University, Maastricht, Netherlands.; 3 Department of Internal Medicine, Maastricht University Medical Centre, Maastricht, Netherlands.; 4 Cardiovascular Research Institute Maastricht (CARIM), Maastricht University, Maastricht, Netherlands.; 5 Department Chrometa, KU Leuven, Leuven, Belgium.; 6 Department of Gastroenterology-Hepatology and Metabolic Centre, University Hospitals Leuven, Leuven, Belgium.; 7 Department of Medicine, University of Cambridge, Cambridge, United Kingdom.; 8 Central Diagnostic Laboratory, Clinical Chemistry, Maastricht University Medical Centre, Maastricht, Netherlands.; 9 Internal Medicine Research Unit, Pfizer Research and Development, Pfizer Inc., Cambridge, Massachusetts, USA.; 10 Department of Radiology and Nuclear Medicine, Maastricht University Medical Centre, Maastricht, Netherlands.; 11 Institute for Clinical Diabetology, German Diabetes Centre, Leibniz Institute for Diabetes Research at Heinrich Heine University Düsseldorf, Düsseldorf, Germany.; 12 German Centre for Diabetes Research, München-Neuherberg, Germany.; 13 Leiden University Medical Centre, Department of Clinical Epidemiology, Leiden, Netherlands.

**Keywords:** Clinical trials, Endocrinology, Metabolism, Carbohydrate metabolism, Drug therapy, Genetic diseases

**To the editor:** Hereditary fructose intolerance (HFI) is an inborn error of fructose metabolism caused by a defect in aldolase B (*ALDOB*). Ingestion of fructose results in rapid accumulation of fructose 1-phosphate (Fru-1P) and depletion of inorganic phosphate (Pi) and ATP in tissues expressing mutant *ALDOB* (enterocytes, hepatocytes, and proximal tubules) (Figure 1A). This is clinically manifested by abdominal pain, nausea, vomiting, hypoglycemia, hypophosphatemia, and proximal tubular dysfunction. Fatal cases of acute liver and renal failure have been described (1).

Currently, a life-long fructose-restricted diet is the only effective treatment for HFI. However, pharmacological inhibition of ketohexokinase (KHK), which would prevent Fru-1P accumulation, may be beneficial for HFI (Figure 1A). Lanaspa and colleagues demonstrated that *Aldob^–/–^* mice were indeed rescued from the toxic effects of fructose when crossed with *Khk^–/–^* mice (2). Therefore, we explored the effects of PF-06835919, a potent, specific, reversible, oral KHK inhibitor, on intestinal, hepatic, and renal fructose tolerance in patients with HFI.

To ensure that PF-06835919 effectively suppresses hepatic fructose metabolism, we first measured in vivo fructose metabolism using ^31^P-magnetic resonance spectroscopy in 14 overweight/obese participants with metabolic dysfunction–associated steatotic liver disease (MASLD) upon PF-06835919 versus placebo treatment (see Supplemental Methods and Supplemental Figure 1A; supplemental material available online with this article; https://doi.org/10.1172/JCI187376DS1). The 60 g oral fructose load did not elicit a hepatic phosphomonoester (PME) peak (reflecting Fru-1P) or a transient decrease in hepatic Pi concentrations after PF-06835919 treatment, as compared with placebo, indicating effective KHK inhibition (Figure 1, B and C). There was no carry-over effect (data not shown).

We subsequently exposed three patients with HFI (Supplemental Table 1) to a series of paired, single-blinded oral glucose and fructose tolerance tests, alternating per day, after prior treatment with PF-06835919. With each consecutive block, the dose of fructose (2.5, 5.0 and 7.5g) controlled with a dose of glucose (matched for sweetness intensity) was increased, depending on tolerability (Supplemental Figure 1B). Results for glucose tests are shown in Supplemental Figure 2.

Patient A reported no intestinal complaints after 2.5 g fructose or glucose equivalent. Urinary fructose already increased after the run-in of PF-06835919 and increased further after the oral fructose load, indicative of KHK inhibition. There were no signs of proximal tubular dysfunction. There was a slight decrease in serum phosphate and glucose and an increase in uric acid. To exclude reduced fasting tolerance as a potential explanation, we repeated the measurements in fasting condition. Glucose and phosphate levels remained stable, whereas serum uric acid increased even further (Figure 1, D–G, and Supplemental Figure 3A). Although complete hepatic KHK inhibition was observed upon PF-06835919 in participants with MASLD and serum uric acid levels did not show a clear pattern in patient A, we cannot fully exclude residual KHK activity as an explanation for the decrease in serum glucose in patient A. Hence, as a safety caution, we decided not to expose this patient to higher fructose doses.

Patient B experienced gastroenteritis during run-in, which in hindsight was already present before PF-06835919 treatment. The study procedure was therefore initiated on a new occasion with sufficient tablets for two blocks of tolerance tests. No gastrointestinal symptoms were reported, nor were there signs of proximal tubular dysfunction upon 2.5 and 5 g fructose (or glucose equivalent). Blood glucose and serum phosphate and uric acid remained fairly stable upon both fructose tests (Figure 1, H–K, and Supplemental Figure 3B).

Patient C was exposed to all three doses of fructose. The dose-dependent increase in urinary fructose excretion was in accordance with patients A and B. No intestinal complaints were reported during any of the fructose or glucose tests. There were no signs of proximal tubular dysfunction. Furthermore, serum phosphate and uric acid and blood glucose levels were stable after all fructose doses (Figure 1, L–O, and Supplemental Figure 3C).

Of note, we did not include a placebo arm, since we considered it unethical to expose untreated HFI patients to fructose, which can already be toxic at low doses. However, as the doses of fructose greatly exceeded their daily intake (and recommended allowance), we believe it unlikely that absence of clinical symptoms in patients upon oral fructose is attributable to a placebo effect. We also observed dose-dependent increases in urinary fructose excretion.

In conclusion, PF-06835919 effectively suppresses hepatic fructose phosphorylation in participants with MASLD. Furthermore, PF-06835919 was well tolerated and improved fructose tolerance in patients with HFI. The current outcomes warrant further study that combines clinical pretesting to assess individual safety with longer follow-up and clinically relevant endpoints.

## Supplementary Material

Supplemental data

ICMJE disclosure forms

Supporting data values

## Figures and Tables

**Figure 1:**
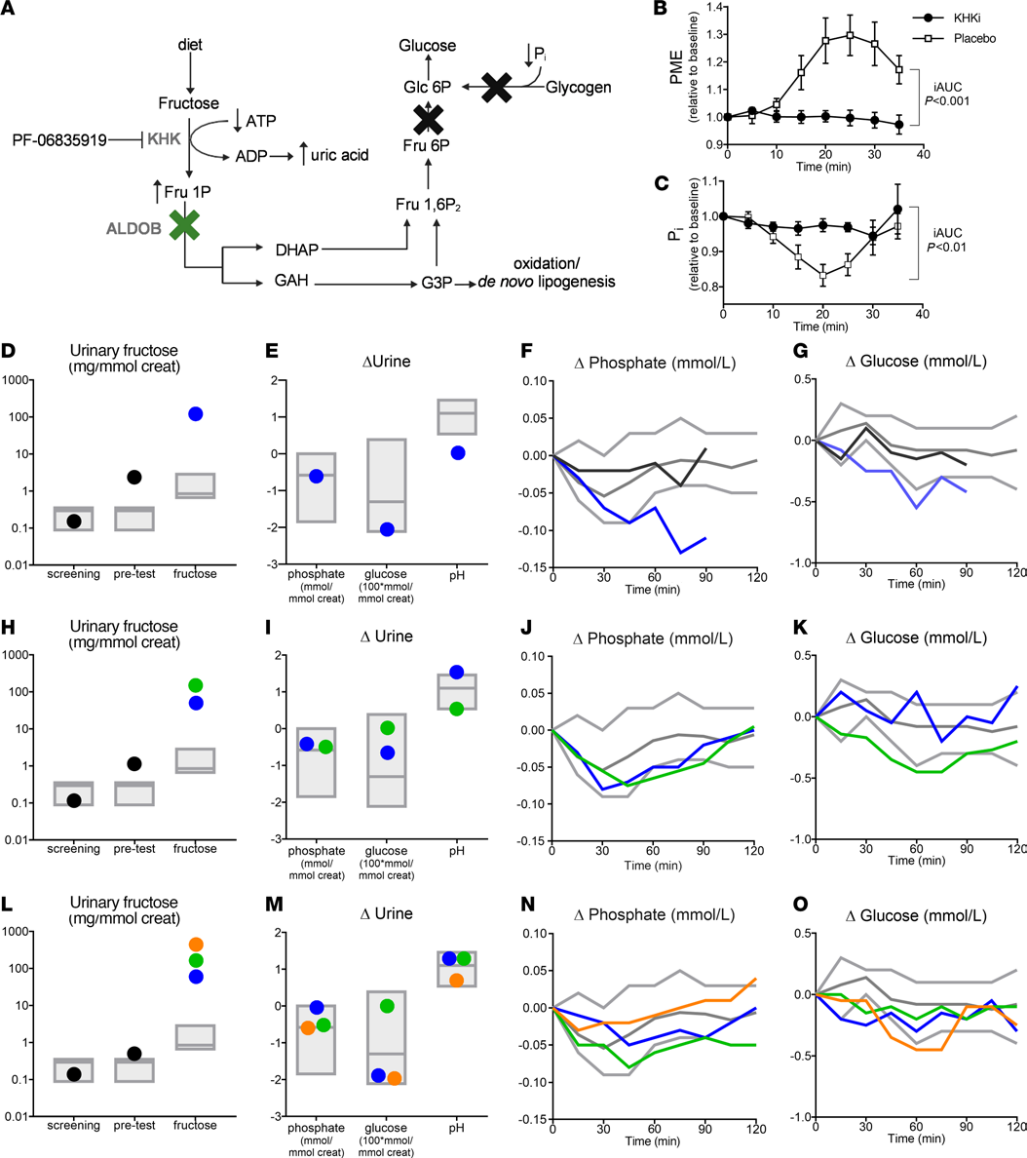
Fructose metabolism in MASLD and fructose tolerance in HFI upon PF-06835919. (**A**) Fructose can serve as a substrate for gluconeogenesis, oxidation, and de novo lipogenesis in the liver. Upon fructose ingestion in HFI, ALDOB deficiency results in the accumulation of Fru-1P and depletion of ATP and Pi. These conditions impair gluconeogenesis and glycogenolysis and consequently induce hypoglycemia. It is hypothesized that pharmacological inhibition of KHK by PF-06835919 will mitigate these biochemical derangements. Created in BioRender. Brouwers, M. (2025) https:// BioRender.com/q91c888. (**B** and **C**) In vivo changes in hepatic PME and P_i_ concentrations in response to a 60-gram oral fructose load after placebo and PF-06835919 treatment in participants with MASLD (*n* = 14). Data are presented as mean ± SEM. (**D**–**O**) Changes in urinary fructose, urinary glucose, phosphate and pH, and serum phosphate and blood glucose after an oral fructose load (2.5 g [blue], 5.0 g [green], 7.5 g [orange]) in patients A (**D**–**G**), B (**H**–**K**), and C (**L**–**O**) treated with PF-06835919. Gray lines represent upper, lower, and mean reference ranges obtained from five healthy individuals (not treated with PF-06835919) after 7.5 g oral fructose. Black lines/dots represent fasted samples.

## Version 1 02/11/2025

In-Press Preview

## Version 2 03/17/2025

Electronic publication
