# Supplementary material for: Safety and efficacy of pharmacological inhibition of ketohexokinase in hereditary fructose intolerance
Source: J Clin Invest. 2025 Feb 11;135(6):e187376. doi: 10.1172/JCI187376 (PMC11910217; doi:10.1172/JCI187376)
Supplement: Supplemental data [file jci-135-187376-s176.pdf]

Index:

Methods

i.	*Sex as a biological variable*	**2**
ii.	*Study approval*	**2**
iii.	*In vivo fructose metabolism by ^{31}P-MRS in PF-06835919-treated patients with MASLD*	**2**
**iv.**	*PF-06835919 in patients with hereditary fructose intolerance*	**4**
v.	*Statistics*	**5**
vi.	*Data availability*	**6**

Author contributions	**6**
References	**6**
Supplementary table 1	**8**
Supplementary figure 1	**9**
Supplementary figure 2	**10**
Supplementary figure 3	**11**

Methods

Sex as a biological variable

Our study included men and women, but the numbers were too small to study any sex-dimorphic effects.

Study approval

Both studies (PF-06835919 in MASLD [NCT05463575] and PF-06835919 in HFI [NCT06089265]) were approved by the Medical Ethical Committee of Maastricht University, and conducted at Maastricht University Medical Centre+. The studies were conducted in accordance with the declaration of Helsinki, and all participants provided written informed consent. Both studies were investigator-initiated and the funder played no role in the study design.

In vivo fructose metabolism by ^{31}P-MRS in PF-06835919-treated patients with MASLD

In a randomized, controlled, double blind cross-over trial, fifteen participants with MASLD (intrahepatic lipid [IHL] content ≥5.56%) received 6 weeks of dosing of PF-06835919 300 mg daily and 6 weeks of dosing with matching placebo (**Figure S1A**). PF-06835919 and placebo tablets (visually identical to PF-06835919) were provided by Pfizer, Inc.. Study medication was packaged in identical bottles, each with a unique number. An independent researcher, not involved in the conduct of this study, received the deblinding key and carried out the controlled randomization using an online randomization tool (randomizer.org).

Previous clinical studies have shown that the 300 mg dose of PF-06835919 results in approximately 90% KHK inhibition in humans, and reduces liver fat in a randomized controlled trials in MASLD (1-3). Participants were recruited through advertisement in and near Maastricht between September 2022 and November 2023. All participants (9 male, 6 female)

had MASLD and were aged between 40 and 75 years (BMI: 31.2 ± 4.7 kg/m^2, Age: 68.7 ± 6.8 years, IHL: 10.7 ± 4.3%). The sample size was calculated on the basis of expected effect size for the change in hepatic insulin sensitivity measured by the hyperinsulinemic euglycaemic clamp, the primary endpoint of this study (which is not the scope of this sub-study). As an explorative objective within the study protocol, *in vivo* fructose metabolism was measured by ^{31}P-MRS on day 41 of the placebo and PF-06835919 arms. This method was based on that described previously by Boesiger et al. (4), but optimized for detection after an oral fructose load instead of intravenous fructose. All MR measurements were performed on a 3T MR system (Achieva 3T-X, Philips Healthcare, Best, Netherlands) with a 14 cm coil (P140-coil, Philips Healthcare, Best, Netherlands), with participants positioned in the prone position. To minimize the signal from the muscles, the power was optimized. This involved a series of free induction decays acquired with a range of flip angles, and the flip angle with the least PCr signal was chosen for the consecutive measurements. To monitor metabolites in time, a series of eight FIDs was acquired using block pulses with the optimized flip angle, with a repetition time of 2000 ms, spectral bandwith of 4000 Hz, an offset frequency of -276 Hz (phosphomonoesters were on resonance), and 144 acquisitions with 2048 data points that were averaged per spectrum. After a baseline measurement, participants consumed an oral 60-grams fructose drink through a straw, whilst remaining in position inside the scanner and afterwards, the remaining 7 spectra of the time series were measured. One participant was excluded from the analysis because he became unwell during the ingestion of the oral fructose. Spectra (from n=14) were analysed by a custom-written MATLAB script, and changes in phosphomonoesters (PME) and inorganic phosphate (Pi) relative to baseline were quantified, and iAUC was calculated (data not shown).

PF-06835919 in patients with hereditary fructose intolerance

In a single arm, open label study, three patients with HFI were treated with PF-06835919 300 mg once daily for 8 consecutive days. This number was deemed sufficient as an exploratory study and feasible given the rare nature of HFI. As noted above, PF-06835919 tablets were provided by Pfizer, Inc. The patients were screened and recruited between June 2023 and November 2023. They were approached for participation by their treating physician and provided written informed consent before any data related to the study were collected. Inclusion criteria were a confirmed diagnosis of HFI and age ≥ 18 years. Exclusion criteria were diabetes mellitus, pregnancy or no use of effective contraception (women), congestive heart failure, severe renal or liver insufficiency, uncontrolled hypertension, or use of drugs that inhibit organic anion transporting polypeptide B1 (OATPB1) transporters.

Daily fructose intake was assessed by a three-day food diary, as described previously (5).

Two days after the start of the study medication (taken in the early morning) patients were exposed to a stepwise increase in oral fructose (2.5, 5.0 and 7.5 grams) to monitor intestinal, renal and hepatic tolerability. As patients with HFI have a natural aversion to sweet tastes – which may elicit symptoms by itself – we also exposed them to oral glucose tests (matched for sweetness to oral fructose: 5.3, 10.5 and 15.8 grams). Patients were blinded to the order of glucose/fructose administration. Each block of oral tests always started with glucose, followed by fructose the next day. This means that the first fructose challenge test was performed after 4 doses of PF-06835919 (**Figure S1B**).

During the tests, abdominal pain and nausea were monitored, starting at T=0, before the oral fructose or glucose load. Abdominal pain was assessed every 15 minutes with a 10-point analogue scale. Nausea was assessed every 5 minutes, where participants indicated if they were less, more or equally nauseous compared to the preceding time point.

Blood was drawn every 15 minutes for 120 minutes to measure blood glucose (Accu-chek Inform II, Cobas, Roche diagnostics, Mannheim, Germany), serum phosphate (enzymatic spectrophotometric assay, Cobas 8000 instrument, Roche Diagnostics, Mannheim, Germany), and uric acid (Cobas Pro analyzer, Roche Diagnostics, Mannheim, Germany) as indicators of hepatic tolerability.

Urine samples were collected before and after the oral tests. Samples were directly analysed for urinary phosphate (enzymatic spectrophotometric assay, Cobas 8000 instrument, Roche Diagnostics, Mannheim, Germany), glucose (enzymatic spectrophotometric assay, Cobas 8000 instrument, Roche Diagnostics, Mannheim, Germany) and pH (Clinitek Novus instrument, Siemens Healthcare Diagnostics, New Orleans, LA, USA), as indicators of proximal tubular dysfunction. Urinary fructose was quantified by Ultra Performance Liquid Chromatography–tandem Mass Spectrometry (UPLC-MS/MS), as described previously (6).

Reference values for normal responses to an oral 7.5g fructose challenge were assessed in five healthy individuals (2 females, 3 males; age range 25- 28 years, BMI range 20-29 kg/m^2), recruited through advertisement. The responses in these individuals are provided as grey lines in **Figure 1D-O, S2 and S3**.

The primary endpoint of this study was fructose tolerance, assessed at the level of the intestines (abdominal complaints), liver (blood glucose and serum phosphate levels) and kidney (urinary pH, glucose and phosphate).

Statistics

Data are presented as mean ± SEM, unless otherwise indicated. To quantify the ^{31}P-MRS signals the trapezoidal rule was used for the calculation of the incremental area under the curve (iAUC). Wilcoxon matched pairs signed-rank tests were used to compare PME and P_i iAUC after placebo and PF-06835919 after a 60-grams oral fructose load. Statistical analyses were

performed using GraphPad Prism 8 and IBM SPSS 24, and a p-value <0.05 was considered statistically significant.

Data availability

Data is available in the supporting data file. Additional data is available upon reasonable request.

Author contributions

M.C.G.J.B, V.B.S, P.S and E.J.C.K. contributed to the conceptualization of the study. M.C.G.J.B, D.C, T.M.C, V.B.S. and E.J.C.K. contributed to the methodology of the study. A.M.B, M.C.G.J.B, J.B, S.J.R.M., V.B.S, J.L.J.M.S, C.G.S. and E.J.C.K. contributed to the collection and analysis of the data. M.C.G.J.B and E.J.C.K. drafted the manuscript and all authors contributed to the intellectual content, review of the manuscript and approved the final version for submission. Authorship order was assigned based on contributions.

References

1. Gutierrez JA, et al. Pharmacologic inhibition of ketohexokinase prevents fructose-induced metabolic dysfunction. *Mol metab.* 2021;48:101196.
2. Kazierad DJ, et al. Inhibition of ketohexokinase in adults with NAFLD reduces liver fat and inflammatory markers: A randomized phase 2 trial. *Med.* 2021;2(7):800-813.e3.
3. Saxena AR, et al. A phase 2a, randomized, double-blind, placebo-controlled, three-arm, parallel-group study to assess the efficacy, safety, tolerability and pharmacodynamics of PF-06835919 in patients with non-alcoholic fatty liver disease and type 2 diabetes. *Diabetes Obes Metab.* 2023;25(4):992-1001.

4. Boesiger P, et al. Changes of Liver Metabolite Concentrations in Adults with Disorders of Fructose Metabolism after Intravenous Fructose by 31P Magnetic Resonance Spectroscopy. *Pediat Res.* 1994;36(4):436-440.
5. Simons N, et al. Patients With Aldolase B Deficiency Are Characterized by Increased Intrahepatic Triglyceride Content. *J Clin Endocrinol Metab.* 2019;104(11):5056-5064.
6. Buziau AM, et al. Development and validation of a UPLC-MS/MS method to quantify fructose in serum and urine. *J Chromatogr B Analyt Technol Biomed Life Sci.* 2020;1155:122299.

Supplementary table 1. HFI patient characteristics at screening

	Patient A	Patient B	Patient C
**Sex**	Female	Male	Male
**Age (years)**	18	27	27
**Mutation**	c.524C>A (p.Ala175Asp)/ c.448G>C (p.Ala150Pro)	c.448G>C (p.Ala150Pro)/ c.448G>C (p.Ala150Pro)	c.448G>C (p.Ala150Pro)/ c.448G>C (p.Ala150Pro)
**BMI (kg/m^2)**	20.0	23.0	20.0
**ASAT (U/L)**	15	15	20
**ALAT (U/L)**	13	16	24
**eGFR ($ml/min/1.73m^2$)**	131	124	111
**Habitual fructose intake (g/day)**	0.6	0.8	1.1
**Age of diagnosis (years)**	2	13	3
**Clinical manifestations at diagnosis**	Vomiting and diarrhoea upon introduction of fruits at weaning	Chronic abdominal complaints, and pallor after fructose containing meals.	Failure to thrive. Vomiting and diarrhoea upon some food products.
**Symptoms upon fructose consumption**	Vomiting and diarrhoea upon small amounts of fructose consumption (e.g. salad dressing or sauces).	Pallor, sweating, dizziness upon small amounts of fructose (e.g. cashew nuts in meals)	Abdominal complaints after small amounts of fructose (food products containing ≥ 2g sucrose/100g; e.g. green asparagus).

*eGFR calculated based on serum creatinine and age (CKD-EPI creatinine equation).

Supplementary Figure 1.

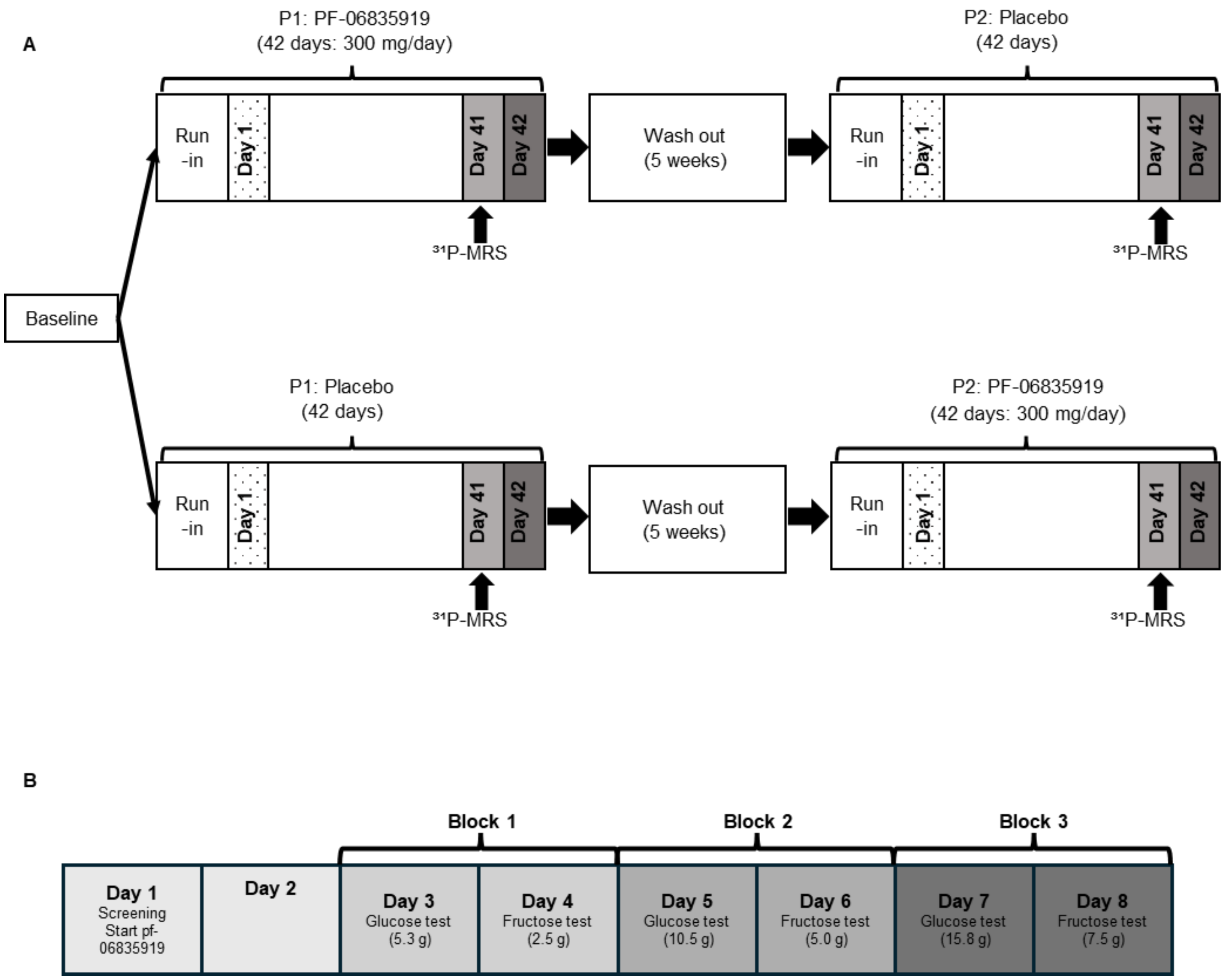

(A) Study design for patients with MASLD, and (B) study design for patients with HFI.

Supplementary Figure 2

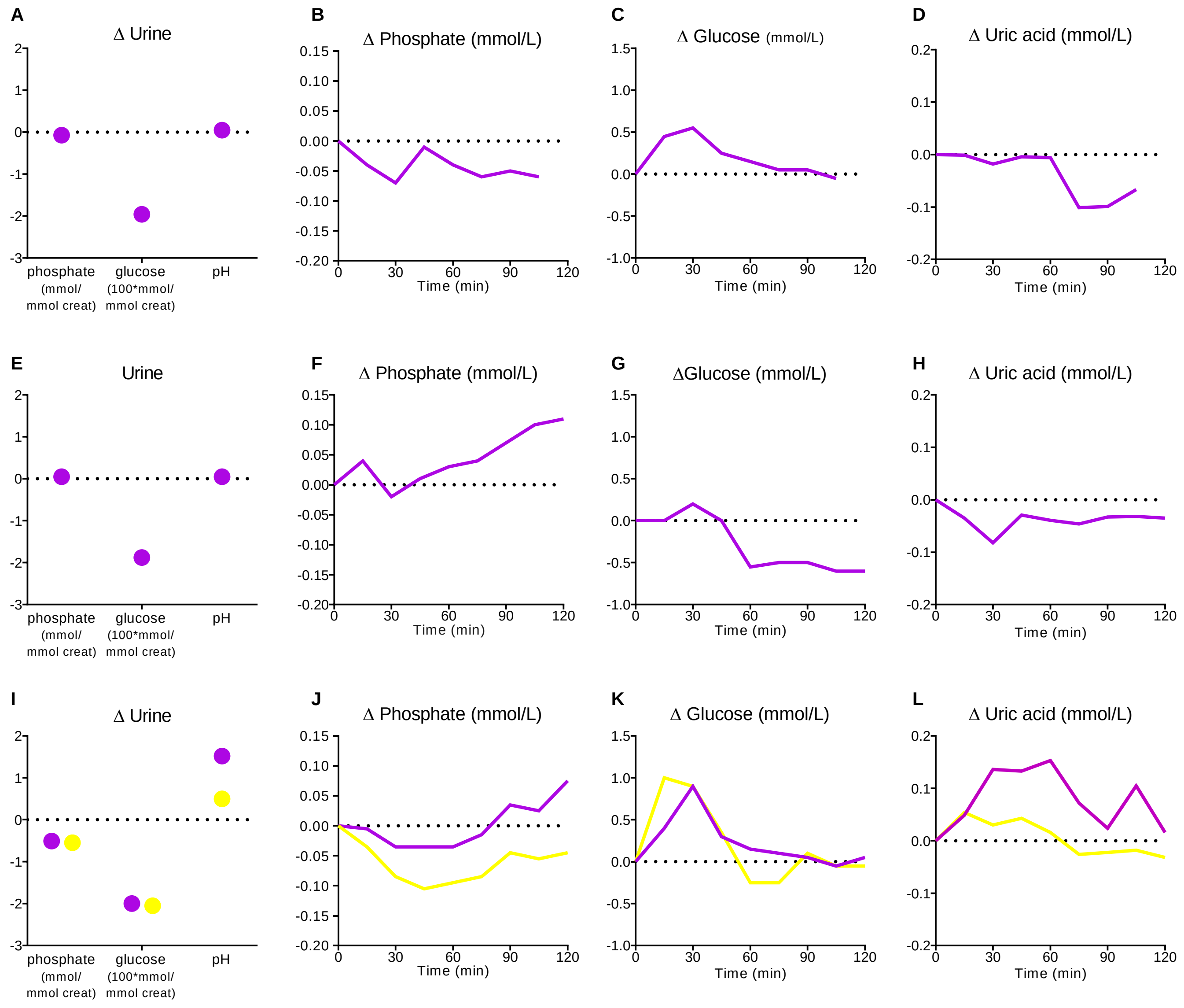

Change in urinary glucose, phosphate and pH, and serum phosphate blood glucose and serum uric acid after an oral glucose load (5.3g [purple], 10.5g [yellow]) in patients A (**A-D**), B (**E-H**) and C (**I-L**) treated with PF-06835919.

Supplementary Figure 3

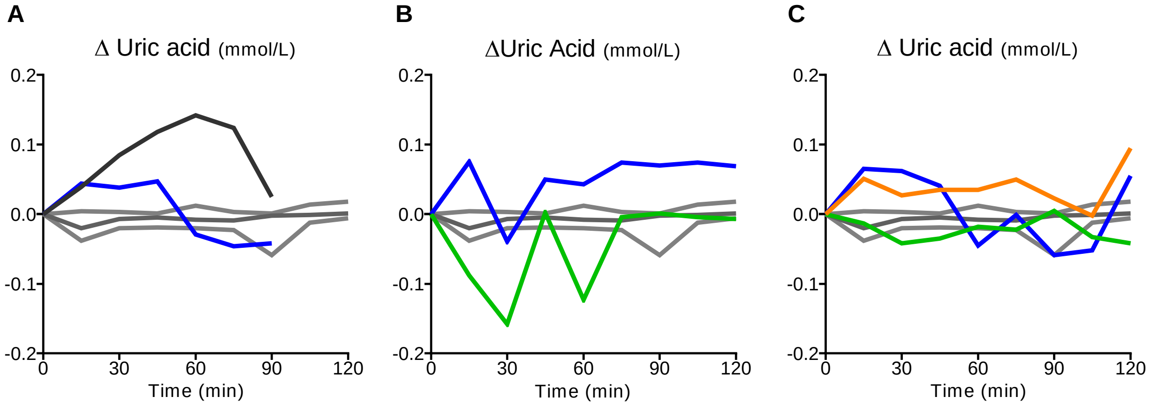

Changes in plasma uric acid levels after an oral fructose load (2.5g [blue], 5.0g [green], 7.5g [orange]) in patients A (**A**), B (**B**) and C (**C**) treated with PF-06835919. Grey lines represent upper, lower and mean reference ranges obtained from five healthy individuals (not treated with PF-06835919) after 7.5g oral fructose. Black lines/dots represent fasted samples.
